# Elevated Basal Pre-infection CXCL10 in Plasma and in the Small Intestine after Infection Are Associated with More Rapid HIV/SIV Disease Onset

**DOI:** 10.1371/journal.ppat.1005774

**Published:** 2016-08-10

**Authors:** Mickaël J. Ploquin, Yoann Madec, Armanda Casrouge, Nicolas Huot, Caroline Passaes, Camille Lécuroux, Asma Essat, Faroudy Boufassa, Béatrice Jacquelin, Simon P. Jochems, Gaël Petitjean, Mathieu Angin, Kathleen Gärtner, Thalía Garcia-Tellez, Nicolas Noël, Thijs Booiman, Brigitte D. Boeser-Nunnink, Pierre Roques, Asier Saez-Cirion, Bruno Vaslin, Nathalie Dereudre-Bosquet, Françoise Barré-Sinoussi, Mathilde Ghislain, Christine Rouzioux, Olivier Lambotte, Matthew L. Albert, Cécile Goujard, Neeltje Kootstra, Laurence Meyer, Michaela C. Müller-Trutwin

**Affiliations:** 1 Institut Pasteur, Département de Virologie, Unité HIV, Inflammation et Persistance, Paris, France; 2 Institut Pasteur, Département de infection et épidémiologie, Unité de Recherche et d'Expertise Epidémiologie des Maladies Emergentes, Paris, France; 3 Institut Pasteur, Département d’immunologie, Unité Immunobiologie des cellules dendritiques, INSERM U818, Paris, France; 4 INSERM CESP U1018, Université Paris Saclay, Université Paris Sud, AP-HP, Le Kremlin-Bicêtre, France; 5 Commissariat à l’Energie atomique et aux énergies alternatives, Division IMVA/IDMIT/IMETI/SIV CEA, Fontenay-aux-Roses, France; 6 INSERM U1184, Université Paris Saclay, Université Paris Sud, Le Kremlin-Bicêtre, France; 7 Service de Médecine interne, Hôpitaux universitaires Paris Sud, Le Kremlin-Bicêtre, France; 8 Academisch Medisch Centrum, Laboratory of Viral Immune Pathogenesis, Amsterdam The Netherlands; 9 Institut Pasteur, Département de Virologie, Unité Régulation des infections rétrovirales, Paris, France; 10 Centre Hospitalier Universitaire Necker–Hôpitaux de Paris, Laboratoire de Virologie, Paris, France; 11 EA7327 Université Paris Descartes, Paris, France; Vaccine Research Center, UNITED STATES

## Abstract

Elevated blood CXCL10/IP-10 levels during primary HIV-1 infection (PHI) were described as an independent marker of rapid disease onset, more robust than peak viremia or CD4 cell nadir. IP-10 enhances the recruitment of CXCR3+ cells, which include major HIV-target cells, raising the question if it promotes the establishment of viral reservoirs. We analyzed data from four cohorts of HIV+ patients, allowing us to study IP-10 levels before infection (Amsterdam cohort), as well as during controlled and uncontrolled viremia (ANRS cohorts). We also addressed IP-10 expression levels with regards to lymphoid tissues (LT) and blood viral reservoirs in patients and non-human primates. Pre-existing elevated IP-10 levels but not sCD63 associated with rapid CD4 T-cell loss upon HIV-1 infection. During PHI, IP-10 levels and to a lesser level IL-18 correlated with cell-associated HIV DNA, while 26 other inflammatory soluble markers did not. IP-10 levels tended to differ between HIV controllers with detectable and undetectable viremia. IP-10 was increased in SIV-exposed aviremic macaques with detectable SIV DNA in tissues. *IP-10* mRNA was produced at higher levels in the small intestine than in colon or rectum. Jejunal IP-10+ cells corresponded to numerous small and round CD68neg cells as well as to macrophages. Blood IP-10 response negatively correlated with *RORC* (Th17 marker) gene expression in the small intestine. CXCR3 expression was higher on memory CD4+ T cells than any other immune cells. CD4 T cells from chronically infected animals expressed extremely high levels of intra-cellular CXCR3 suggesting internalization after ligand recognition. Elevated systemic IP-10 levels before infection associated with rapid disease progression. Systemic IP-10 during PHI correlated with HIV DNA. IP-10 production was regionalized in the intestine during early SIV infection and CD68+ and CD68neg haematopoietic cells in the small intestine appeared to be the major source of IP-10.

## Author Summary

Chronic immune activation is a hallmark of HIV infection and contributes in multiple ways to HIV persistence. Here, we gained insights on the association between a pro-inflammatory chemokine, CXCL10/IP-10 and HIV infection in four cohorts of HIV+ individuals, studied at distinct stages of infection (before, primary and chronic stage with spontaneous- and treatment-controlled infection). We further analyzed pathogenic and non-pathogenic SIV infections to address IP-10 levels and the presence of infected cells in tissues (lymph nodes, small and large intestine). We found that elevated systemic IP-10 levels before HIV-1 infection associate with a more rapid disease progression. During primary infection, IP-10 in blood strongly correlated with the amount of infected cells in blood. The animal model showed that IP-10 expression was regionalized in the intestine and highest in the small intestine. Studies of aviremic animals suggest that high IP-10 is indicative of viral replication in lymphoid tissues. Haematopoietic cells rather than epithelial/endothelial cells mainly contributed to the IP-10 production in small intestine (jejunum). The receptor of IP-10 was highly expressed on memory CD4+ T cells, i.e. major target cells for the virus. This study contributes to our understanding of the establishment of HIV reservoirs and why IP-10 associates with HIV/AIDS.

## Introduction

Chronic immune activation is a hallmark of HIV infection [1]. Effective combined-antiretroviral therapy (cART) reduces HIV viremia to undetectable levels, but milder chronic immune activation nonetheless persists and is associated with onset of both AIDS and non-AIDS illnesses [2, 3]. The mechanisms fuelling chronic inflammation in HIV infection are poorly understood and probably multifactorial. Translocation of microbial products from the gastrointestinal tract may be an important driving factor [4–6, 7, 8].

Studies of SIV+ non-human primates (NHP) such as Asian macaques (MAC) and natural hosts of SIV such as African green monkeys (AGM) support a role of immune activation and microbial translocation in HIV pathogenesis [1, 5, 6, 7, 8–15]. SIV infection in natural hosts is characterized by high viral load but does not result in chronic inflammation [1, 15, 16]. Strong inflammatory responses are only transient in natural hosts and by the end of the primary phase of infection, they are dampened to pre-infection levels [1, 9–15]. We thus asked whether HIV-infected individuals with only weak inflammation near the end of primary HIV infection (PHI) have better outcomes [17]. High inflammation level at Fiebig stages III and IV of PHI was indeed associated with rapid loss of CD4+ T-cells. Among 28 pro-inflammatory factors tested, CXCL10/IP-10 was a strong and independent predictive marker of rapid CD4^+^ T-cell loss [17]. During PHI, IP-10 was even a more robust predictive marker than viremia or the CD4^+^ T-cell nadir.

Many cells can produce and release IP-10 [18]. During HIV-1 infection, circulating myeloid cells are the main source of IP-10 in blood [19]. In secondary lymphoid organs from SIV+ macaques, IP-10 is mainly produced by CD3+ T-cells, but also by CD14+ and CD3-CD14- cells [18, 20, 21]. IP-10 is a pro-inflammatory chemokine and a ligand of CXCR3. As CXCR3^+^ CD4^+^ T-cells are the main cellular targets of HIV [22, 23], it is conceivable that IP-10 enhances the trafficking of HIV target cells to lymphoid tissues, thereby promoting new rounds of infection and helping to establish viral reservoirs [24].

Here we attempted to gain further insights into the role of IP-10 during HIV-induced inflammation and its relationship with the levels of infected cells.

## Results

### Pre-existing elevated IP-10 levels are associated with rapid loss of CD4 T cells upon HIV-1 infection

**Fig 1:**
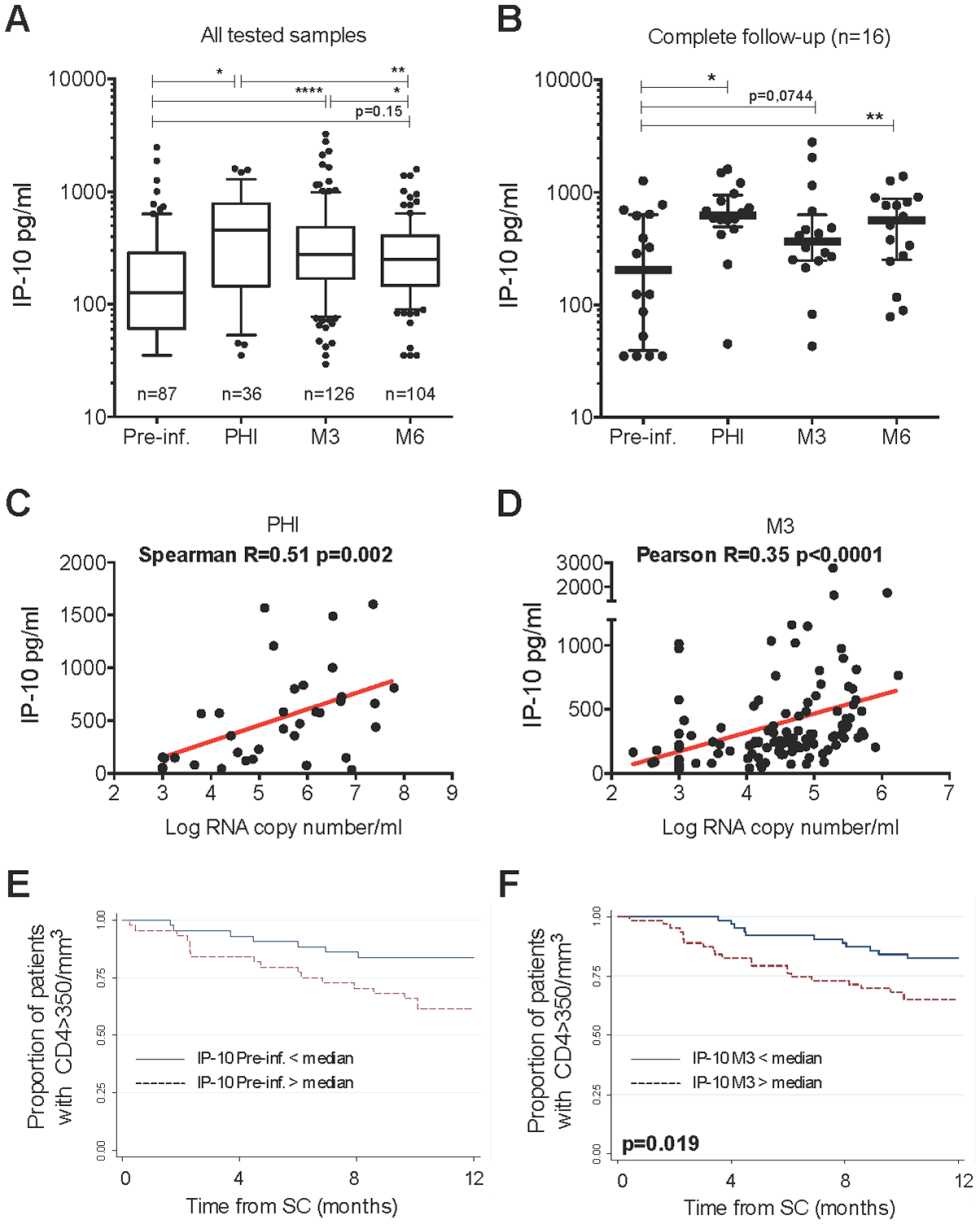
CXCL10/IP-10 dynamics in blood before HIV-1 infection and during the early infection, and its impact on the CD4 T-cells count. IP-10 levels were determined in sera from HIV+ individuals enrolled in the Amsterdam Cohort Studies on HIV/AIDS. **A.** Plasma IP-10 levels. The numbers of patients per group are indicated. **B.** Longitudinal follow-up of the same 16 individuals. **C.** Positive correlation between IP-10 concentrations and viremia at PHI. **D.** Positive correlation between IP-10 concentrations and viremia at M3 post SC. **E.** Impact of pre-infection blood IP-10 concentrations on the CD4 T-cells count after seroconversion. **F.** Impact of M3 blood IP-10 concentrations on the CD4 T-cells count after seroconversion. **E** and **F**: Kaplan Meier survival analysis. Pre-inf. = Pre-infection (median 11 months before seroconversion), PHI = Primary HIV-1 infection, M3 and M6 = 3 and 6 months post seroconversion. * p<0.05, ** p<0.001, **** p<0.00001

We raise the hypothesis that IP-10 attracts target cells for HIV. First, we tested if IP-10 pre-infection levels impact on infection outcome. We quantified IP-10 in blood from 136 patients before HIV infection, during PHI, 3 months (M3) after seroconversion (SC) and/or 6 months (M6) after SC in the Amsterdam cohort (ACS) (characteristics described in Table A in S1 Text). Plasma IP-10 levels were higher during PHI (p<0.05) than before infection, and remained elevated at M3 and M6, albeit at lower levels (p<0.01) (Fig 1A). Similar IP-10 profiles were observed in the subset of 16 patients from whom samples were available at every time point (Fig 1B). IP-10 levels correlated negatively with the CD4 T-cells count [r = -0.19 (p = 0.04) and r = -0.39 (p<0.001) at M3 and M6, respectively], and positively with viremia in PHI (Fig 1C), at M3 (Fig 1D).

Strikingly, elevated IP-10 levels before infection were associated with rapid progression (OR = 3.24 p = 0.01) (Table B in S1 Text), CD4+ T-cell counts falling below 350/mm^3^ more rapidly in individuals with pre-infection IP-10 levels above the median (Fig 1E). In contrast, CD4 T-cell counts before infection had no impact on the rate of progression. Of note, only IP-10 levels measured less than 24 months before infection had such an impact: IP-10 levels measured between 24 and 60 months before infection did not influence the rate of CD4 T-cells loss. IP-10 concentrations in blood before infection were not correlated to canonical immune activation markers (Table C in S1 Text).

Elevated IP-10 levels at M3 post-SC were also associated with rapid CD4+ T-cell decline to below 350/mm3 (Fig 1F) and with an increased risk of rapid progression toward AIDS (OR = 2.54 p = 0.02) before any treatment (Table B in S1 Text). However, at M3, the CD4 T-cell count was a more robust predictor of rapid progression than were IP-10 levels, while both the IP-10 level and the CD4 T-cell counts were more robust predictors than viremia (Table B in S1 Text).

**Fig 2:**
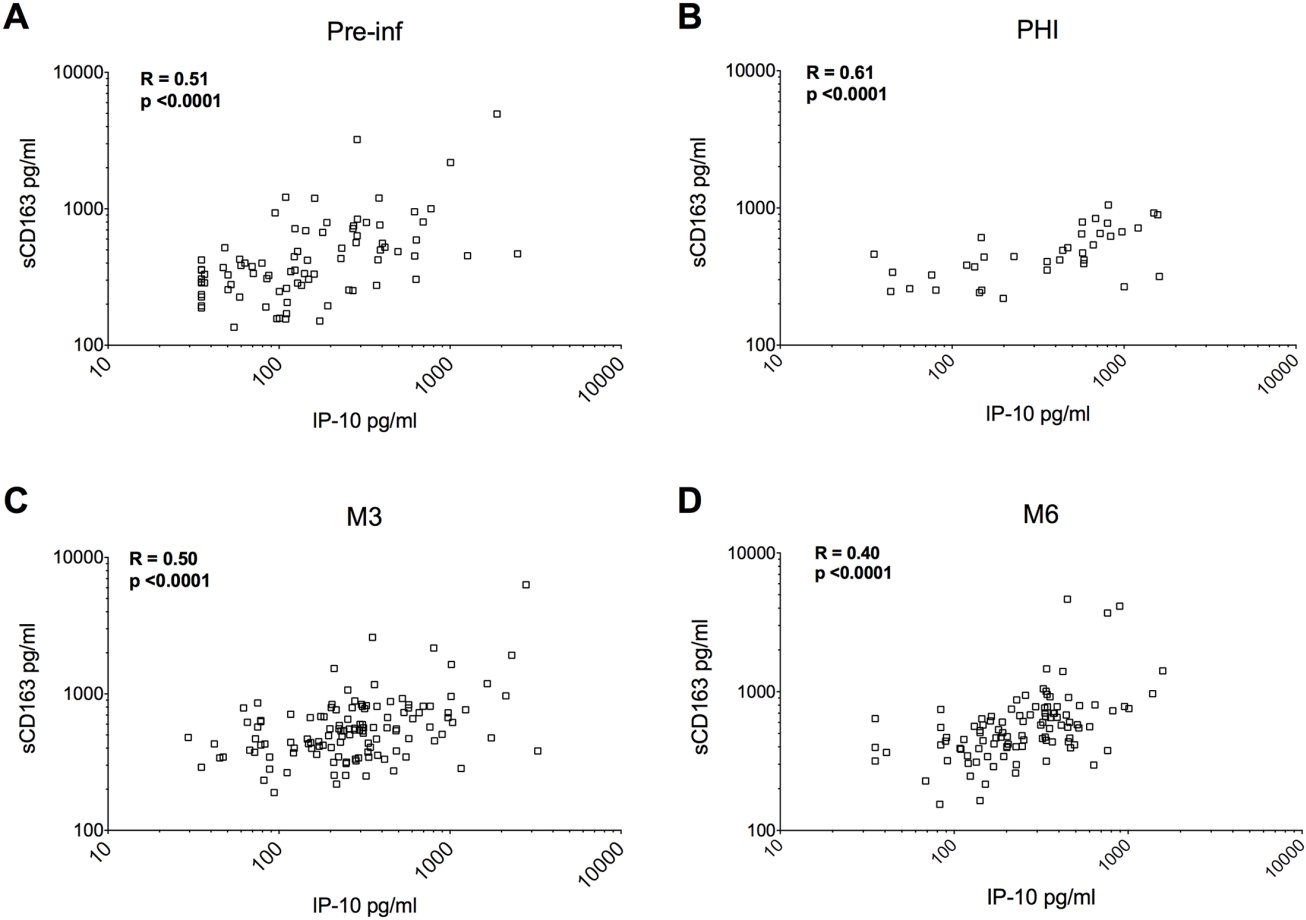
Relationship between blood IP-10 levels and sCD163 during the early stages of HIV-1 infection. Here are displayed the correlations between IP-10 concentrations and sCD163 concentrations before infection (**A**), during PHI (**B**), at M3 (**C**) and at M6 (**D**). PHI = primary HIV infection (M0), M3 = 3 months post alleged date of seronconversion, M6 = 6 months post alleged date of seronconversion in the Amsterdam Cohort (ACS). Pearson R coefficient of correlation and p values are shown on each plot.

In order to compare the robustness of IP-10 in predicting rapid disease onset to other systemic markers of HIV-induced immune activation, we also measured soluble CD163 (sCD163), a monocyte-macrophage activation marker associated with disease progression in HIV+ individuals [25, 26]. sCD163 concentrations in blood were strongly correlated to IP-10 concentrations at all times studied here (Fig 2). However, sCD163 concentrations before and after infection failed to be associated with rapid disease progression (Table B in S1 Text), in contrast to IP-10.

Thus, by analyzing the time course of IP-10 levels, starting before infection, we found that they rose markedly upon HIV infection. Strikingly, pre-existing elevated IP-10 levels were associated with rapid CD4 T-cells loss upon HIV-1 acquisition.

### Blood IP-10 levels correlate with cell-associated viral DNA during primary HIV-1 infection

**Fig 3:**
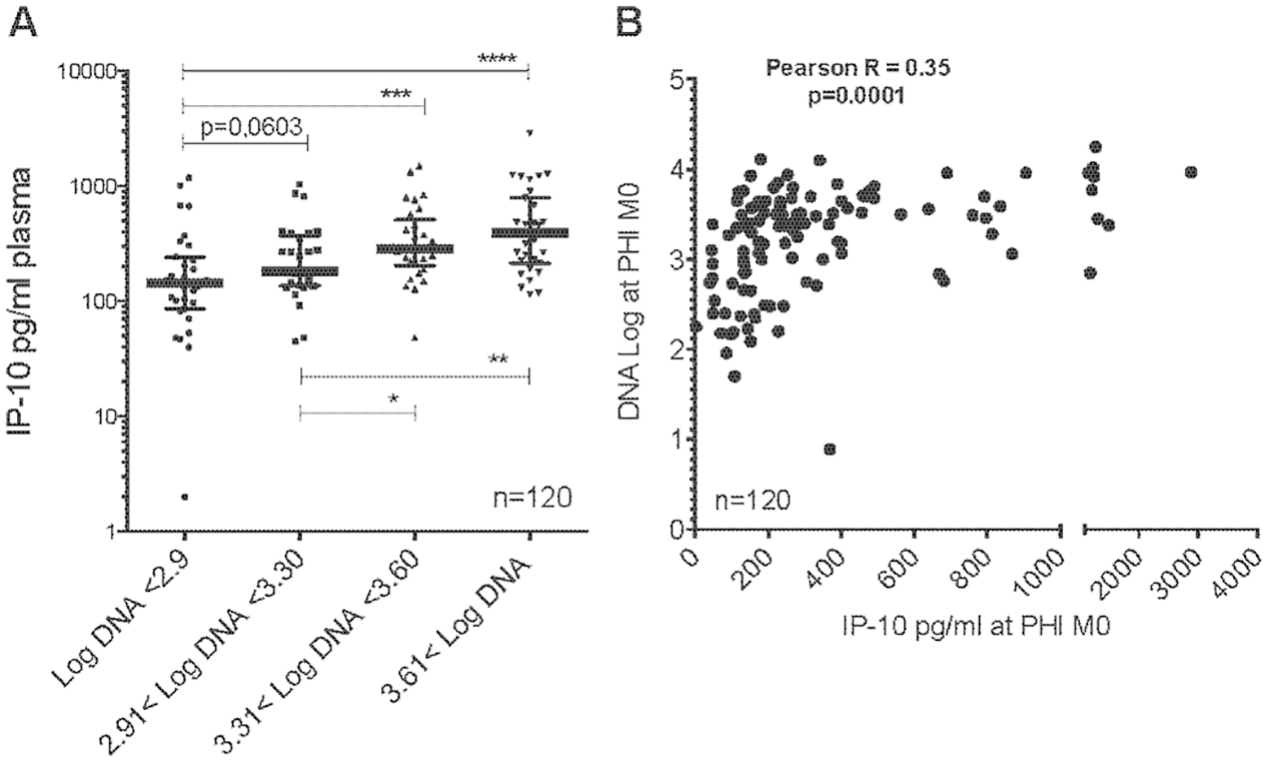
Relationship between plasma IP-10 levels and cell-associated DNA levels during primary HIV-1 infection. **A.** IP-10 plasma concentrations stratified on cell-associated DNA levels during PHI (M0). **B.** Correlation between IP-10 concentrations and cell-associated DNA levels during PHI (M0). M0 = primary HIV-1 infection and time of inclusion (Fiebig stage III/IV).

To further test the hypothesis that IP-10, by promoting CXCR3^+^ cell trafficking, attracts HIV target cells and thereby increases the number of infected cells, we examined the relationship between the IP-10 level and the number of infected cells. We measured cell-associated total viral DNA in order to include both latently infected cells and cells supporting active viral replication. These HIV DNA levels in PBMC from 134 subjects with PHI from the ANRS PRIMO cohort have previously been reported [17]. IP-10 levels strongly correlated with cell-associated HIV-1 DNA (Fig 3).

We also compared the levels of 27 additional inflammatory molecules with the amount of infected cells in the same patients. These markers had been previously analyzed [17]. Among the 27 markers, only IL-18 also positively correlated with total HIV DNA in PBMC (r = 0.3 p = 0.045; Table D in S1 Text), but to a lesser extent than IP-10 (r = 0.35 p<0.0001, Fig 3B). RANTES was the only factor, which had a negative association with total HIV DNA (r = -0.4 p = 0.007; Table D in S1 Text). Altogether Blood IP-10 levels positively correlate with cell-associated viral DNA during in PHI.

### IP-10 levels during controlled viremia

**Fig 4:**
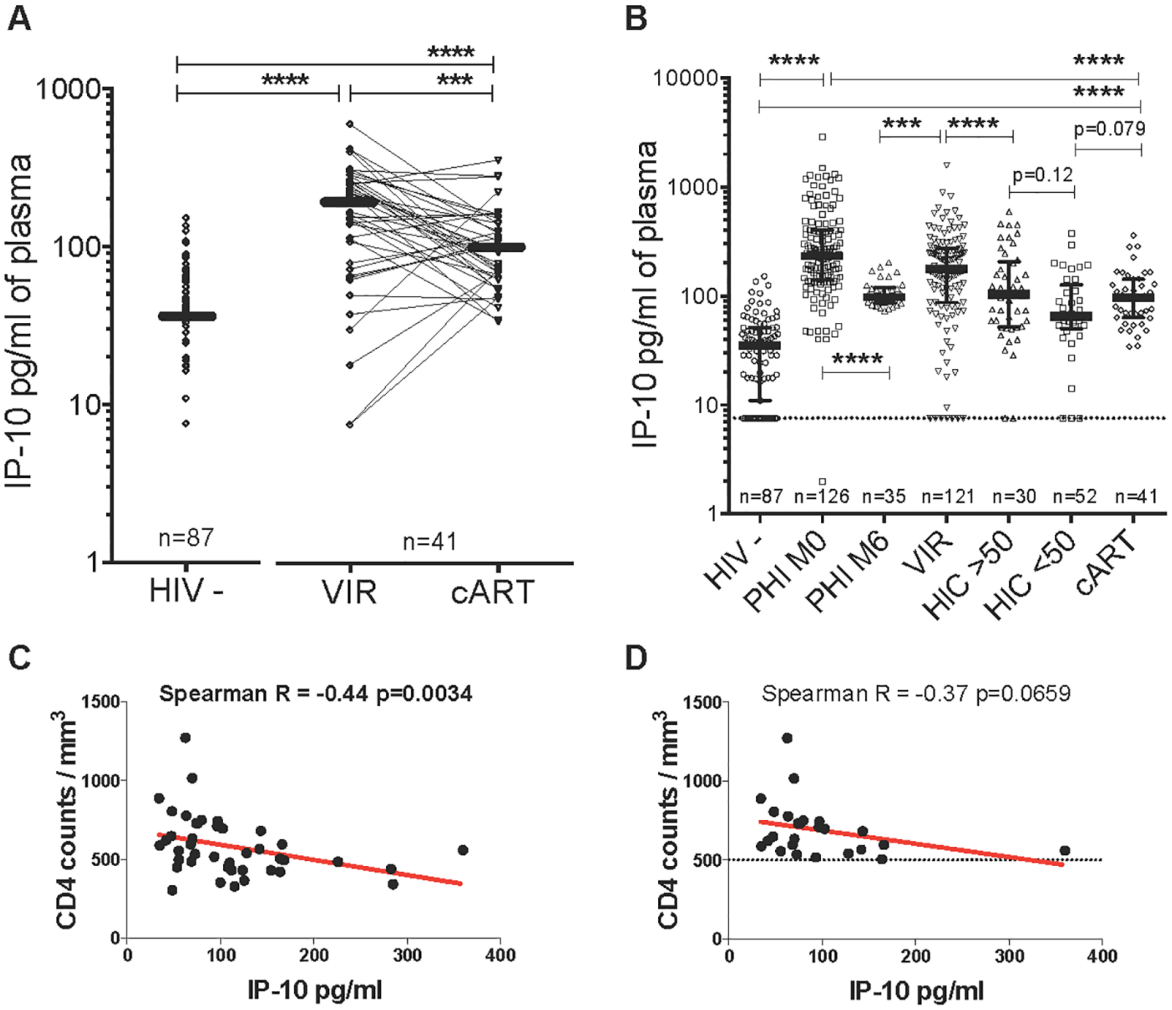
Blood IP-10 levels in patients with controlled HIV viremia. **A.** IP-10 levels were determined in plasma in a longitudinal study, before treatment initiation and after >24 months of antiretroviral treatment, in comparison to HIV-negative individuals. The median (IQR) IP-10 values were 35.3 (10.9–51.6), 190.2 (78.3–269.7) and 97 (63.4–141.7) in HIV-, cART-naïve (VIR) and cART-treated individuals, respectively. IP-10 levels fell in cART-treated patients. B. Comparison of IP-10 levels during primary (PHI), post-acute (M6) and chronic HIV-1 infection (HIC, VIR) by comparison with cART-treated patients. HIC<50 and HIC>50 respectively represent HIV controllers with HIV RNA < and > 50 copy numbers/ml. **C.** Correlation between plasma IP-10 levels and CD4 cell counts during treatment. **D.** Correlation between plasma IP-10 levels and CD4 counts during treatment, considering only individuals with CD4>500 (25 patients). BL = baseline levels before antiretroviral treatment, cART = combination antiretroviral treatment, HIV- = HIV negative, PHI M0 = Primary HIV-1 infection time of enrollment in the PRIMO cohort, M6 = 6 months post M0/PHI, VIR = viremic cART-naïve patients, HIC = HIV controllers, ***p<0.0001, ****p<0.00001. n = number of individuals.

We then examined IP-10 levels in various groups of patients with controlled viremia (Fig 4A and 4B). We first studied HIV controllers (n = 82), divided into two groups: individuals with strong viral control at the time of IP-10 assay (<50 copies of viral RNA/ml) and individuals displaying a moderate viral “blip” at this time point (>50 copies). IP-10 levels in all 82 controllers together were similar to those in the patients on successful cART. However, IP-10 levels tended to be lower in the HIV controllers with <50 copies/ml than in the HIV controllers with >50 copies/ml and cART-treated patients.

Next, we evaluated IP-10 before and during cART (n = 41) (Table E in S1 Text). IP-10 levels fell significantly during cART as compared to pre-treatment levels, but remained higher than in uninfected controls (Fig 4A). Before treatment, IP-10 levels correlated positively with viremia (r = 0.32 p<0.001) and negatively with the CD4 T-cell counts (r = -0.26 p = 0.003). During cART, IP-10 levels correlated negatively with the CD4 T-cell counts (Fig 4C), as strongly as in viremic patients. The recent TEMPRANO and START trials demonstrated that AIDS and non-AIDS events can occur even in patients with high CD4 counts (>500/mm3) [27]. When we considered only patients with CD4 T-cells counts above 500/mm3, there was still a trend towards a negative correlation between IP-10 and CD4 T-cell counts (Fig 4D).

### IP-10 levels in SIV-exposed aviremic macaques

To further determine the relationship between IP-10 and viral replication in a context of viral control, we quantified IP-10 in SIVmac-exposed aviremic macaques. We chose a non-human primate model because it allowed us to investigate viral load in tissues. We studied 34 MAC exposed to moderate doses of SIVmac251. Twenty-seven MAC displayed an expected viremic phenotype. From 3 of these viremic animals, we FACS-sorted the LN cells into four subsets: CD4+ and CD4- T cells expressing or not CXCR3 (Figure A in S1 Text). The viral DNA copy numbers in CD4+ LN cells were high as expected (Figure A in S1 Text). No significant difference could be observed between CXCR3+ and CXCR3neg CD4+ T cells. The samples that were positive for viral DNA were also those positive for *IP-10* gene expression (Figure A in S1 Text).

Surprisingly, 7 of these 34 animals remained aviremic (< 12 copies of SIVmac RNA/ml) during the chronic phase of infection (follow up until 1 year p.i.), despite the fact that the viral doses used resulted in 100% of infection in previous experiments [28]. None of the aviremic animals seroconverted during follow-up. We quantified total SIV DNA in total LN cells from the SIV-exposed aviremic macaques. PCR amplification of cell-associated DNA on total LN cells was negative in 5 of the 7 aviremic animals, while positive (4–35 copies/10^6^ cells) in 2 animals (AX414 and 30845) on day 14 p.i.

**Fig 5:**
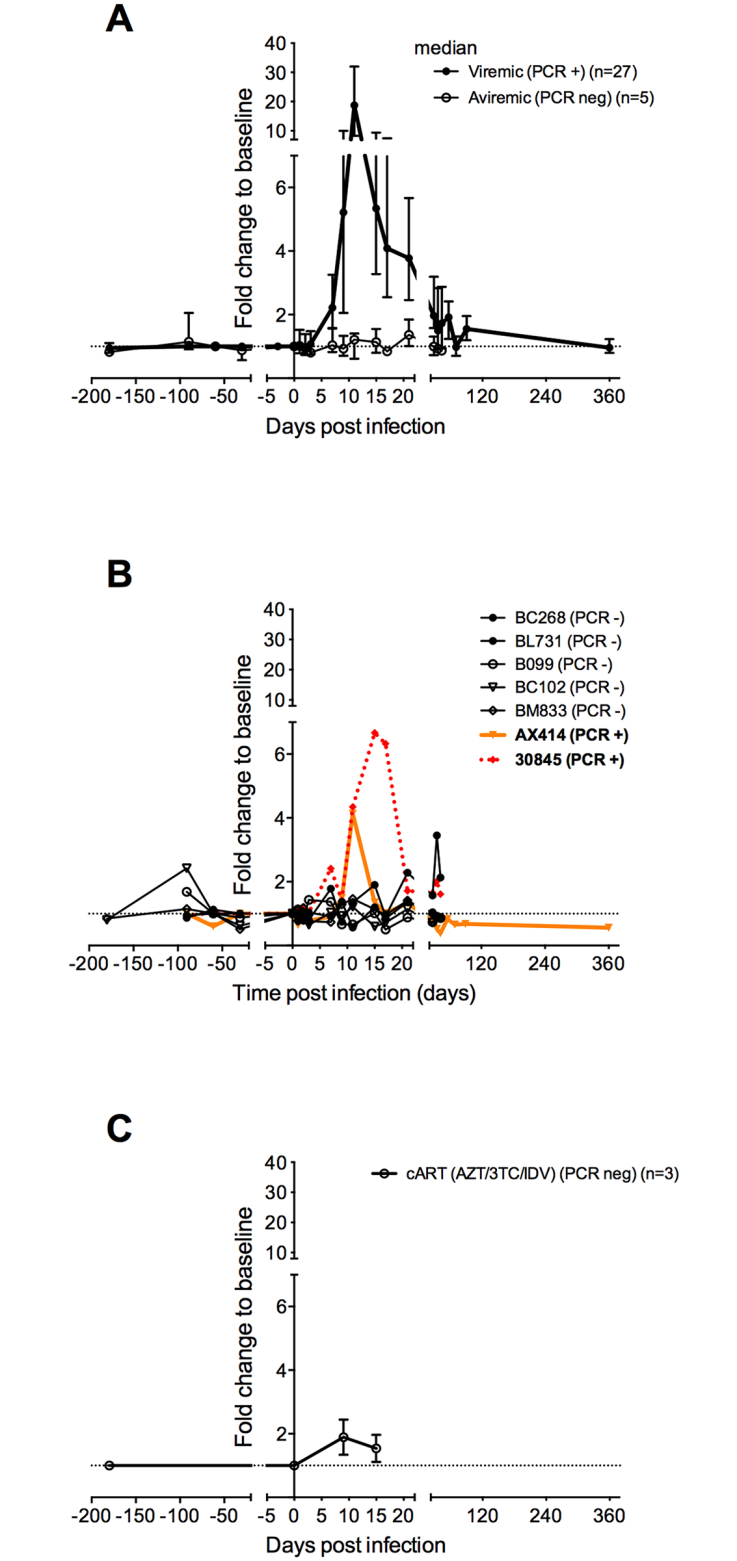
Dynamics of IP-10 levels in viremic and aviremic animals. IP-10 levels in plasma were determined in a longitudinal study, before infection and after SIVmac251 challenge in cynomolgus macaques. Values are expressed as the fold change from baseline (median of 3 to 4 values obtained before infection). **A. IP-10** dynamics in 27 viremic and 5 SIV-exposed aviremic animals are shown (median and interquartile range). The latter had no detectable SIVmac DNA in lymph nodes. **B.** Blood IP-10 dynamics in 2 aviremic animals with detectable cell-associated DNA in lymphoid tissues. Animals AX414 and 30845 were inoculated intrarectally with 5 and 50 AID_50_ of SIVmac251, respectively. PCR+ = detectable lymph node cell-associated SIVmac DNA on day 14 p.i., PCR neg = undetectable lymph node cell-associated SIVmac DNA.

We then compared plasma IP-10 dynamics in three groups of animals (viremic, aviremic with or without detectable viral DNA in tissues). IP-10 levels were elevated in viremic animals, as expected (Fig 5A). Viremia and IP-10 levels correlated positively with one another (r = 0.69, p<0.0001). In the 5 aviremic animals with no detectable viral DNA in tissues (LN), IP-10 levels remained low during primary infection (Fig 5B). In contrast, IP-10 levels increased in the 2 animals (AX414 and 30845) with detectable SIV DNA during primary infection (Fig 5B). We then compared levels in the aviremic animals with those in 3 MAC in which cART was initiated 4 h after infectious challenge [29]. The animals were treated until day 14 p.i. and then sacrificed. None of these animals had detectable viral DNA in lymphoid tissues during follow-up [29]. These cART-treated animals displayed a weaker induction of IP-10 during follow-up as compared to viremic animals (Fig 5C).

Together, these observations support a strong association between blood IP-10 levels and active viral reservoirs in lymphoid tissues.

### High expression of CXCR3+ on memory CD4+ T cells

**Fig 6:**
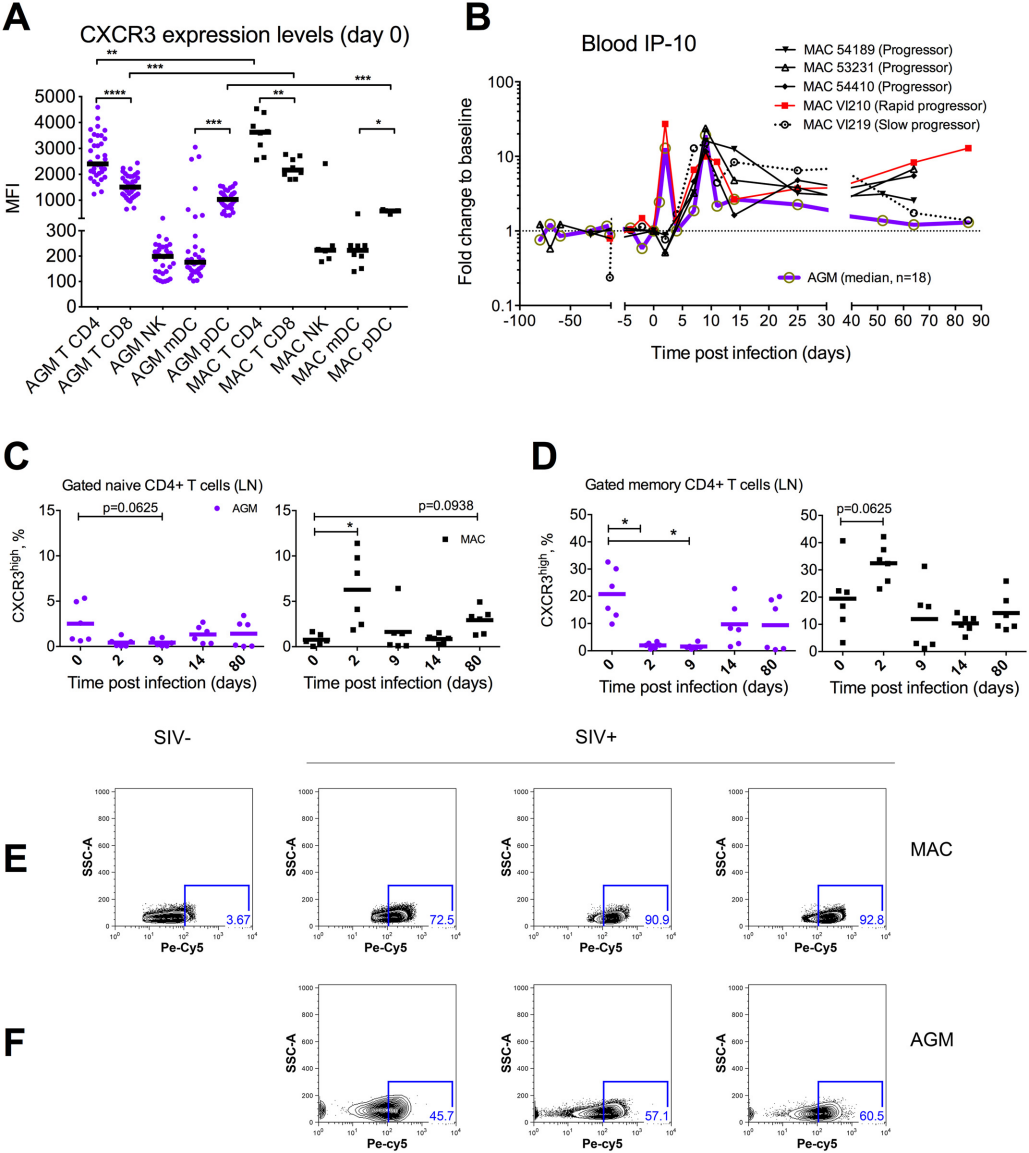
Dynamics of CXCR3+ T-cells and blood IP-10 levels during pathogenic and non-pathogenic SIV infections. **A.** CXCR3 expression levels as mean intensity of fluorescence (MIF) in rhesus macaques (n = 8) and AGM (n = 18) peripheral blood cell subsets before SIV inoculation. **B.** Blood IP-10 dynamics in 5 rhesus macaques *versus* AGMs (n = 18, median is shown here) IP-10 levels in plasma were determined in a longitudinal study, before infection and after SIVmac251 or SIVagm.sab92018 challenge in macaques or in AGMs, respectively. IP-10 values are expressed as the fold change from baseline (median of 3 to 4 values obtained before infection). **(C-F).** CXCR3 expression dynamics from SIVmac infected cynomolgus macaques (n = 3–6) and from SIVagm-infected AGMs (n = 3–6) **C.** Dynamics of CXCR3 expression in LN naïve CD4+ T cells upon SIV infection. **D.** Dynamics of CXCR3 expression in LN memory CD4+ T cells upon SIV infection. (**E-F**). Flow cytometry analysis of internalization levels of CXCR3 in lymph node CD4 T cells in cynomolgus macaques (n = 4) and in African green monkeys (n = 3). Plots of scatter measurements and CXCR3 staining are shown here. Each plot represents an animal.

Since IP-10 triggers the trafficking of cells expressing its ligand, CXCR3, we sought to address the dynamics of CXCR3+ CD4+ T cells *in vivo* in parallel to the IP-10 response upon SIV infection. We first determined the levels of CXCR3 expression by flow cytometry on distinct haematopoietic cells in blood before SIV infection in rhesus macaques and AGMs. Strikingly, CXCR3 was mostly expressed on CD4+ T cells when compared to other immune cells (Fig 6A). CXCR3 expression was also significantly higher on CD4+ T cells than on CD8+ T cells.

We then assessed the dynamics of CXCR3+ within the naïve (CD28+ CD95neg) and memory (CD28+CD95+ and CD28-CD95+) CD4+ T cells in LN. This memory cell fraction typically contains the vast majority central memory CD4+ T cells. We found that the frequency of CXCR3+ cells was higher within the memory subset than within naïve CD4+ T cells. Both in macaques and AGM, these cell subsets quickly exhibited changes in their frequencies upon infection (Fig 6C and 6D). The frequencies of CXCR3+CD4+ T cells tended to increase at day 2 p.i. in macaques (p = 0.0625). In the contrary, AGMs exhibited decreased frequencies of CXCR3-expressing cells in memory CD4+ T cells at days 2 and 9 p.i. (p = 0.0313) (Fig 6C and 6D).

All AGMs and one macaque displayed a peak of IP-10 at days 2 and 9 p.i. (Fig 6B). We therefore wondered whether decreases in CXCR3 expression could be related to internalization of the receptor as described for the IL-7R in HIV/SIV infections [30]. To address this hypothesis we evaluated the rate of CXCR3 internalization in LN CD4 T cells from 3 SIVmac-infected macaques and 3 SIVagm-infected AGMs (Fig 6E and 6F). In both species, we observed in the infected animals high proportion of LN CD4 T cells having internalized CXCR3. The levels of CXCR3 in the permeabilized cells varied between 72 and 92% in macaques and between 45 and 60% in AGMs.

Altogether, CXCR3 was mostly expressed on CD4+ T cells when compared to other immune cells and within CD4+ T cells, more frequently expressed on memory than on naïve cells. Modulations in the frequencies of CXCR3+ CD4+ T cells were observed upon infection. Significant decreases might in part be explained by internalization of the CXCR3 receptor in response to the increased production of its ligands (IP-10 or other CXCR3 ligands) during infection.

### Regionalization of IP-10 expression in the small intestine during SIVmac infection

To determine the origin of IP-10 in blood, we measured IP-10 expression in the largest lymphoid tissue: the intestine. The latter is also the largest site of HIV/SIV replication. We analyzed intestinal IP-10 production in 5 SIVmac-infected rhesus macaques. We also compared the IP-10 expression pattern in a non-pathogenic model (5 SIVagm-infected AGMs). AGMs were used because they are known to display a high level of viral replication in the gut, in the absence of chronic inflammation [7, 31]. Furthermore, as the intestine is regionalized into several sections with specific functions [32], and as we found higher infection rate of the small intestine than in the large intestine in SIVmac-infected cynomolgus macaques (Figure B in S1 Text), we analyzed the IP-10 gene expression in 4 distinct sections, i.e. jejunum, ileum, colon and rectum.

**Fig 7:**
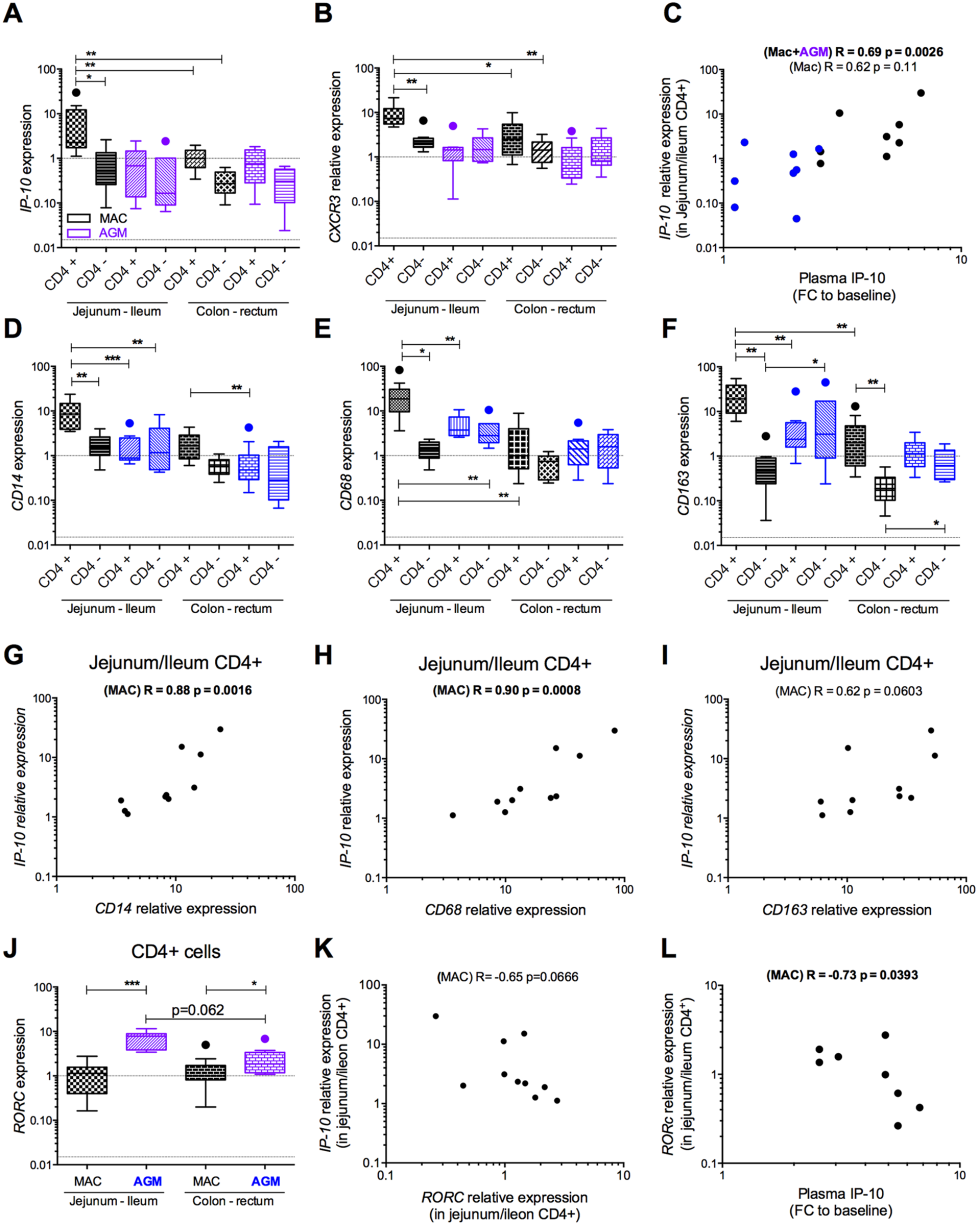
Cellular gene expression profiles in the intestine. **A-L.** Cellular gene expression levels were evaluated in CD4neg and CD4pos leukocytes from 4 distinct segments of the intestine of SIV+ non-human primates, i.e. rhesus macaques (n = 5) and African green monkeys (n = 5) on day 65 p.i. Raw values are represented. **A**. *IP-10* gene expression. **B.** *CXCR3* gene expression. **C.** Correlation between blood IP-10 levels and *IP-10* gene expression in the upper intestine. *IP-10* gene expression levels were normalized to expression in the rectum of each animal. IP-10 levels in blood are expressed as the fold change from baseline. (**D-F**) *CD14*, *CD68* and *CD163* gene expression levels were evaluated in CD4neg and CD4+ leukocytes from different segments of the digestive tract of SIV+ non-human primates obtained at necropsy (day 65 p.i.). **G.** Correlation between *CD14* and *IP-10* gene expression levels in the small intestine. **H.** Correlation between *CD68* and *IP-10* gene expression levels in the small intestine. **I.** Correlation between *CD163* and *IP-10* gene expression levels in the small intestine. **J.** *RORc* gene expression levels in CD4+ leukocytes from the small intestine. **K.** Correlation between *RORc* and *IP-10* gene expression levels. **L.** Correlation between blood IP-10 levels and *RORc* gene expression levels in the small intestine. MAC = SIV-infected rhesus macaques (black), AGM = SIV-infected African Green Monkeys (blue).

During SIVmac infection, *IP-10* was strongly expressed by CD4^+^ cells in the small intestine (jejunum/ileum) (Fig 7A), and more strongly than by CD4- cells in the small intestine or both CD4^+^ and CD4^-^ cells in the large intestine. No such regionalization of IP-10 was seen in AGMs. Likewise, *CXCR3* expression was the strongest in CD4+ cells of the small intestine during SIVmac infection, whereas this profile was not observed in AGMs (Fig 7B). *IP-10* expression correlated with *CXCR3* expression (r = 0.66, p = 0.0009).

*IP-10* expression levels in CD4+ cells of the small intestine correlated with plasma levels when the two species were grouped for analysis (Fig 7C). When we considered only MAC, which reduced the statistical power, there still was a trend towards a positive correlation (Fig 7C). No such correlation was found in the colon/rectum.

Thus, the strongest *IP-10* expression was detected in the CD4+ fraction in the small intestine in SIVmac-infected macaques. Moreover, IP-10 levels in plasma tended to correlate with those in the small intestine.

### Cellular sources of IP-10 expression in the gut

We then sought to determine which CD4+ cell subset was responsible for elevated IP-10 expression in the small intestine. It has been described that IP-10 is produced by monocytes and macrophages during HIV/SIVmac infection [19, 33–35]. We therefore quantified the expression of genes associated with macrophages (*CD14*, *CD68* and *CD163)* in the same samples as those analyzed above for *IP-10* mRNA. All three macrophage markers (*CD14*, *CD68* and *CD163)* were more strongly expressed in small-intestinal CD4+ leukocytes from MAC than from AGM, or in any other gut section from MAC (Fig 7D–7F). *IP-10* expression levels correlated strongly with these gene expression levels (Fig 7G–7I).

**Fig 8:**
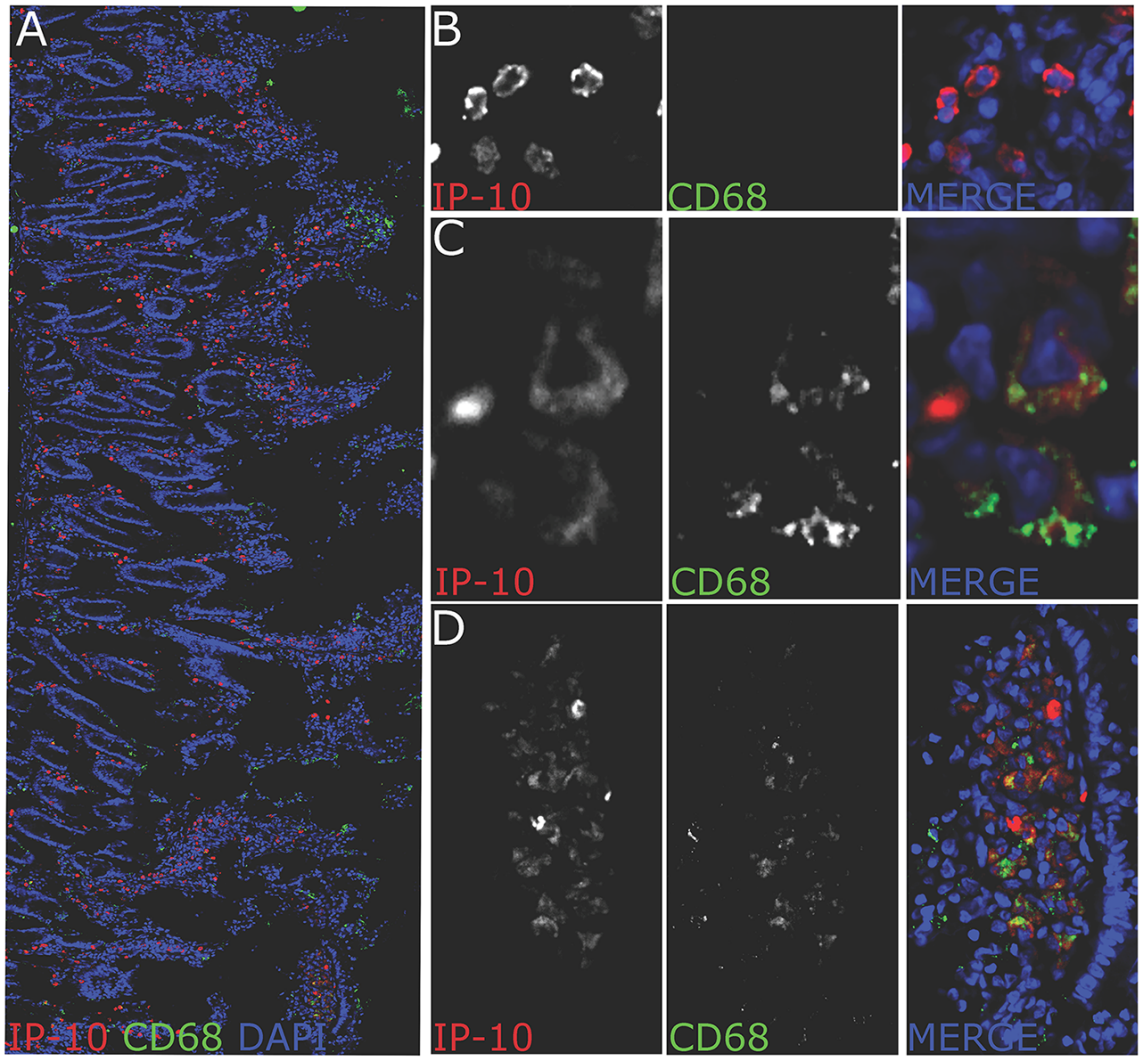
IP-10 positive cells in jejunum of chronically SIVmac-infected macaques. (a) confocal image of a jejunum section stained for CD68 (green), IP-10 (red) and total nucleus (blue). The picture represents the distribution of IP-10+ cells in jejunum of chronically infected macaques (240 dpi) (representative picture for one animal out of 3 animals studied). IP-10+ cells were found in the lamina propria around and in the villi as well as at the top of the villi. Most IP-10+ cells were CD68 negative. A fraction of CD68+ cells produced IP-10. Cells were often organized in clusters. (B) Magnification showing IP-10+ cells negative for CD68 found at the bottom of the villus. The morphology evoke T cells. (C) Magnification showing IP-10+ positive for CD68+. (D) Magnification showing IP-10+ cells organized in a cluster. Many IP-10+ cells in these clusters were CD68+. Pictures were obtained using a leica SP8 confocal microscope. All pictures were analyzed with ImageJ software.

To address whether IP-10 is produced at the protein level in gut macrophages, we performed immunohistochemistral stainings in jejunum fragments of macaques harvested at necropsy (day 240 p.i). IP-10 expression was detected throughout the jejunum in cells that had a shape and localization distinct from epithelial/endothelial cells (Fig 8A). We found a massive expression of IP-10 from small and round CD68neg cells, which ressembled in their shape lymphocytes (Fig 8B) and to a lesser extent from large CD68+ cells (Fig 8C). Very often CD68+IP-10+ cells were found in clusters at top of villi (Fig 8D). These data demonstrate that IP-10+ is produced by macrophages in the jejunum. However many smaller, CD68neg cells produced IP-10 as well.

### Strong *IP-10* expression in the small intestine correlates with the loss of *RORC*-expressing CD4^+^ cells

To further analyze the link between IP-10, macrophages and intestinal inflammation, we quantified the expression of ISGs such as *MX1* and *IFI30*. IP-10 expression was positively correlated to *MX1* and *IFI30* gene expression. (Figure C in S1 Text). Although these ISGs were also correlated with the macrophage markers, these correlations were less robust than with IP-10 (Figure C in S1 Text).

During HIV-1/SIVmac infections, mucosal immunity in the intestine is generally gradually impaired, notably with a characteristic loss of Th17 cells, which is absent in natural hosts of SIV [5, 36, 37]. As a read-out for gut damage, we quantified the expression of *RORC*, the master transcription factor for Th17 cells, in enriched CD4+ leukocytes from the intestinal sections. We detected stronger *RORC* expression in CD4+ leukocytes from AGM than MAC (Fig 7J).

*RORC* and *IP-10* expressions correlated negatively with one another in small-intestinal CD4+ leukocytes (r = -0.74, p = 0.0034) from all the studied NHP. There was also a trend towards a negative correlation when we considered only MAC (Fig 7K). A negative correlation between *RORC* in the small intestine and plasma IP-10 (Fig 7L) was observed.

Pathogenic SIVmac infection is characterized by a skewed Th response towards a Th1 phenotype in the gut at the detriment of Th17 cells, in contrast to natural hosts [38–40]. To determine if this negative correlation between *IP-10* and *RORC* were associated with a particular profile of Th differentiation in the analyzed CD4+ cell population, we went on evaluating the *IP-10* expression in Th subsets. We sorted circulating primary human Th subsets. We observed that primary human circulating Th17 cells are devoid of a strong *IP-10* gene expression in sharp contrast to primary Th1-like cells which express the highest levels of transcriptional *IP-10* gene activity (Figure D in S1 Text). Altogether these data indicate that IP-10 is associated with inflammation in the small intestine and suggest a negative association between the levels of IP-10 and Th17 cells.

## Discussion

Here, we studied the relevance of IP-10 as a marker of disease progression before infection, and examined why IP-10 is so strongly associated with HIV pathogenesis.

We found that pre-existing elevated IP-10 levels were associated with an increased risk of rapid CD4+T-cells loss upon HIV infection in the ACS. Elevated levels of IP-10 prior to infection may be multifactorial: (i) resulting from co-infections with viruses although we excluded co-infections with HIV-2, HBV and HCV but we couldn’t exclude a co-infection with TB [41]. Indeed this latter report showed that during reactivation of latent TB infection IP-10 is found at elevated concentrations in blood. We couldn’t completely rule out (ii) elevated immune activation [42]. However, when we analyzed additional immune activation/inflammation markers, such as Ki-67, CD8+DR+CD38+, CD8+CD70+, CD4+DR+CD38+, CD4+CD70+, before infection, we couldn’t see any significant impact of such markers on the rate of CD4+ T cells upon HIV-infection and none of them were significantly correlated to IP-10 pre-infection levels in blood (Table C in S1 Text). The caveat was the very low number of patients with documented immune activation markers before infection.

In addition, we analyzed sCD163 levels. They were significantly correlated to those of IP-10 but sCD163 levels failed to predict rapid disease onset in contrast to IP-10 pre-infection levels.

Lower IP-10 levels had been reported in the genital mucosa of highly HIV-exposed-seronegative women than in HIV-seronegative and -seropositive women [43, 44]. Blood IP-10 levels were recently reported to be higher in transmitting HIV-1–infected individuals and in their HIV-1–seroconverting partners than in HIV-1–infected and uninfected partners [45]. This suggested that elevated IP-10 increased the risk of HIV-1 acquisition. It is possible that this increased risk of HIV-1 acquisition, and the more rapid CD4+ T-cells loss observed here in individuals with higher IP-10 levels before infection, is due to IP-10 enhancement of the infection. We indeed found a strong correlation between IP-10 and the amount of cell-associated HIV DNA. Recently we have analyzed plasma IP-10 levels in the ANRS OPTIPRIM study, where patients received mega-ART therapy starting from PHI [46]. IP-10, but not IL-6, sCD14 nor sCD163 was positively correlated with blood HIV-DNA at inclusion (r = 0.53, p = 0.018) and only IP-10 levels among 5 inflammatory markers assessed in plasma correlated with total HIV DNA in semen (A. Cheret, personal communication). In our animal models, IP-10 levels helped to discriminate between SIV-exposed aviremic animals with and without detectable tissue infection. Several studies have shown that elevated IP-10 levels contribute to excessive recruitment of CXCR3+ T-cells into lymphoid tissues during pathogenic SIVmac infection [47, 48]. This was also described in important sites of viral entry, notably the foreskin of sexually active men with a high risk of acquiring HIV [49]. CXCR3 expression is highest in activated memory CD4+ T-cells [22]. It is possible that IP-10 attracts not only CXCR3+ immune cells with potential antiviral activity but also major HIV target cells, indirectly enhancing viral dissemination and the establishment of viral reservoirs. Recent studies have shown a high infection rate among CXCR3+ CD4+ T-cells [22], which are also preferentially enriched for HIV DNA in HIV-infected individuals on cART [23]. IP-10 has also been shown to enhance the susceptibility of resting naïve CD4 T-cells to HIV infection [50]. Here we observed distinct dynamics of CXCR3+ CD4 T cells on LN from SIV-infected macaques and natural hosts. These increased frequencies of CXCR3+ memory CD4 T cells in macaque LN versus reduced frequencies of CXCR3+ memory CD4 T cells in AGM LN during the acute phase of infection may be due to mobilization of these cells in the context of a strong pro-inflammatory context (CXCR3-ligands) in macaques. We could not see such dramatic increases in AGM and we can’t exclude that the CXCR3 was either internalized or that the CXCR3+ memory CD4+ T cells are migrating to another tissue in natural hosts. In macaques, we didn’t detect a higher rate of infection in CXCR3+ versus CXCR3neg CD4+ cells. As CXCR3 might be dramatically internalized upon recognition of its ligands during infection (Fig 6E and 6F), the interpretation of the viral distribution in the context of increased production of IP-10 (and other CXCR3 ligands) *in vivo* is complex. More animals and more studies need to be done at critical time points and in animals with distinct levels of CXCR3 ligands. Though, when we looked at the intestinal mucosa, we found a significant higher rate of infection in the small intestine where the expression of IP-10 and CXCR3 were the highest. Overall, our findings further demonstrate that elevated IP-10 levels are a strong predictive marker of disease progression but raise the question whether it might, directly and/or indirectly, also promote the establishment of viral reservoirs.

Our data in animals suggest that IP-10 in plasma derives in large part from the gut, the largest lymphoid tissue. Unexpectedly, the *IP-10* response was regionalized in macaques, being higher in the small intestine than the large intestine. *CXCR3* expression showed the same regionalization. Previous studies have shown marked regional variations in the abundance of infected cells in the gut, but most focused on specific compartments and not the entire gut [51, 52]. In cART-treated patients, the small intestine also seems to harbor more active viral reservoirs than the periphery or the rectum [53, 54]. Here we also observed a higher infection rate in the small intestine than in the large intestine, correlating with the IP-10/CXCR3 regionalized expression. Further, *IP-10* mRNA expression in MAC small intestine positively and strongly correlated with the expression of macrophage-associated markers (*CD14*, *CD68* and *CD163* mRNA). This association can have several explanations. It might have an indirect cause as both IP-10 and macrophage levels in the gut might be the consequence of higher inflammation. It might also be explained by a higher number of macrophages in the gut or finally by an increase in production of IP-10 in the gut. IP-10 levels correlate with expression of the activation markers CD11b and CD38 on monocytes [55]. In the latter report, the sCD14 levels did not correlate with any of these molecules. The same authors suggest that IP-10, but not sCD14, is a robust and easier tool to measure monocyte activation [55]. Further, an accumulation of CD68+ and CD163+ macrophages in the duodenal mucosa of HIV-infected patients was reported in parallel to increased levels of pro-inflammatory molecules such as IP-10 [20]. By studying patients in the COPANA cohort, we found that plasma IP-10 correlated with the concentrations of monocytic activation markers sCD163 (r = 0.57 p<0.001), sCD14 (r = 0.35 p = 0.008) and TFN-α (r = 0.43 p<0.001), but not with CRP, IL-6, MCP1, sTNFR1 or sTNFR2 (Figure E in S1 Text). Infiltration of the small intestine (duodenum) of HIV-infected individuals by inflammatory CD68+/CD163+ macrophages has been reported in HIV+ individuals in absence of cART [20]. Thus, elevated IP-10 production in the small intestine might derive from infiltrating activated macrophages during progressive HIV/SIV infections. The *IP-10*, *CD14*, *CD68* and *CD163* gene expression in the small intestine of SIVmac-infected rhesus macaques seem to be in line with these observations in HIV+ individuals. We show here that macrophages are indeed one important source of IP-10 production in the jejunum SIV-infected rhesus macaques (Fig 7). This is consistent with previous reports in humans. During acute HIV-1 infection, IP-10 production in blood was found to be associated with circulating myeloid cells [19]. However, many other cells produced IP-10 as well, consistent with data in lymph nodes [18, 20, 21]. Indeed, IP-10 was also more often confined to CD68neg small and rounded-shape cells (Fig 8). These might include predominantly CD4+ lymphocytes since *IP-10* mRNA expression levels where highest in the CD4+ cell fraction. However, we cannot exclude differences in the profiles of mRNA and cellular sources due to fact that the PCR and IHC analyses were performed in distinct animals and distinct time points (Day 65 p.i and D240 p.i., respectively). Finally *IP-10* increases might result from both increases in cell numbers as well as from intra-cellular upregulations. Altogether, we determined the cellular source of IP-10 and demonstrate a regionalization of IP-10 production in the gut.

The small intestine in particular is remarkably enriched in IL-17 producing T cells [56]. A gradient of T-cell IL-17 production has indeed been reported along the intestinal tract, with the small intestine being enriched in such lymphocytes [56]. We found that IP-10 levels in both blood and the small intestine correlated negatively with the presence of Th17 in the small intestine, suggesting that blood IP-10 levels mirror the extent of gut damage. *RORC* expression in the small intestine was stronger in AGMs than in MAC, which supports the relevance of our model. We found that *IP-10* mRNA expression in MAC small intestine negatively correlated with the presence of gut Th17 cells (Fig 7). IP-10 expression might be indirectly associated to intestinal inflammation and gut damage. Our observations from primary human Th subsets clearly confirm a confinement of IP-10 gene expression in genuine Th1 cells rather than Th17. Thus the negative correlation between IP-10 and RORC in our study could be due to the biased Th1 response at the detriment of Th17 response, which has been described in pathogenic SIVmac infection in contrast to natural hosts of SIVs [38–40].

Persistently moderate IP-10 levels were observed in HIC, as reported elsewhere [57, 58]. IP-10 seems to distinguish between HIC who experience viral blips (Fig 3B) and rapid loss of CD4+ T-cells [57, 58], and could prove useful for identifying those patients with controlled viremia, including HIC, who need therapeutic interventions to further delay disease progression.

In summary, this study of IP-10 in four cohorts of HIV-infected patients and in two non-human primate models provides new information on the tissue source of this pro-inflammatory mediator, reveals its regionalization in gut and indicates an association with the cell infection rate during HIV-1 infection.

## Methods

### Ethics statement

Studies were conducted with ethical agreements and with the informed consent of each patient. Patient enrollment respects European guidelines and established guidance promulgated by the World Medical Association in its declaration of Helsinki. All patients were adult subjects. The scientific board of the Amsterdam cohort studies and of the French ANRS cohorts PRIMO C06, COPANA C09 and CODEX C21 approved this study. Institut Pasteur “Comité de recherché Clinique” CoRC #2013–05 approved this study.

Animals were housed in the facilities of the CEA (“Commissariat à l'Energie Atomique”, Fontenay-aux-Roses, France) IDMIT facilities (Center for Infectious Disease Models and Innovative Therapies), Fontenay-aux-Roses, France (permit number A 92-032-02) or the Pasteur Institute, Paris, France (permit number A 78-100-3). All experimental procedures were conducted in strict accordance with the European guideline 2010/63/UE for the protection of animals used for experimentation and other scientific purposes (French decree 2013–118) and with the recommendations of the Weatherall report. The monitoring of the animals was under the supervision of the veterinarians in charge of the animal facilities. All efforts were made to minimize suffering, including efforts to improve housing conditions and to provide enrichment opportunities (e.g., 12∶12 light dark schedule, provision of monkey biscuits supplemented with fresh fruit and constant water access, objects to manipulate, interaction with caregivers and research staff). All procedures were performed under anesthesia using 10 mg of ketamine per kg body weight. For deeper anesthesia required for lymph node removal a mixture of ketamine and xylazine was used. Paracetamol was given after the procedure. Euthanasia was performed prior to the development of any symptoms of disease (e.g., for macaques when the biological markers indicated progression towards disease, such as significant CD4^+^ T cell decline and increases of viremia). Euthanasia was done by IV injection of a lethal dose of pentobarbital.

### Patients and samples

A large serum library derived from cART-naïve patients is available, including pre-infection samples from many patients enrolled in the Amsterdam Cohort Studies (ACS) of HIV infection and AIDS. None of the 136 subjects studied here had started antiretroviral therapy when samples were collected. These patients had an estimated date of seroconversion (SC) defined as the midpoint between the date of the last visit with a negative HIV test and the first visit with a positive HIV test (complete or incomplete western blot) [59]. None of the 136 subjects had started antiretroviral therapy when samples were collected. Patients were categorized as rapid progressors (RP) or slow/normal progressors as previously described [17]. Basically, rapid progressors had CD4 T cell counts below 350/ml 12 months post-SC. PHI (M0) was defined by an incomplete WB, with detectable HIV RNA load and/or p24 (Fiebig stage III/IV). Samples collected before infection were obtained at least 3 months before the estimated date of SC. Patients co-infected with other bloodborn pathogens (HIV-2, HBV, HCV) were excluded.

The viremic cART-naive subjects (VIR, n = 121) were part of the French ANRS C09 COPANA cohort (See supplementary materials and Table C in S1 Text). A subgroup of 41 patients was submitted to cART (>24 months on cART and >12 months with VL < 50 copies/mL).

The HIV controllers (HIC, n = 82) were enrolled in the French ANRS C021 CODEX (See supplementary materials and Table C in S1 Text). The samples collected at PHI M0 (n = 126) and PHI M6 (n = 35) were from subjects enrolled in the French ANRS C06 PRIMO cohort, who are thoroughly described in [17].

The viremic cART-naive subjects (VIR, n = 121) were part of the French ANRS C09 COPANA cohort. The main objective of this ongoing cohort created in 2004 is to prospectively evaluate the impact of HIV infection and ART on morbidity and mortality in recently diagnosed (<1 year) HIV-1-infected cART-naive adults in France. The Paris-Cochin Ethics Committee approved the study protocol and all the participants give their written informed consent. Among the 800 patients enrolled in the COPANA cohort, 214 joined the metabolic sub-study [60]. The 121 VIR patients corresponded to patients with available CRP, IL-6, MCP1, TNFα, sCD14, sCD163, sTNFR1 and TNFR2 values and who initiated cART at enrollment or during follow-up. The 121 VIR patients were enrolled within a median of 6 months (range 3.8–9.1) after HIV-1 diagnosis. IP-10 was measured at cART initiation. A subgroup of 41 patients on sustained successful cART (>24 months on cART and >12 months with VL < 50 copies/mL) was identified. They were enrolled in the COPANA cohort less than 6 months after diagnosis of HIV infection. None of the subjects enrolled in our study received immunosuppressive drugs, IFN therapy or chemotherapy, and none had cancer, autoimmune diseases or HIV-unrelated chronic inflammatory metabolic disorders at enrollment. The HIV controllers (HIC, n = 82) were enrolled in the French ANRS C021 CODEX cohort with their written informed consent. This French multicenter cohort was created in 2009. To be enrolled, patients had to be diagnosed as HIV-infected for more than 5 years, to remain cART-naive with viremia below 400 copies/ml in five consecutive assays, regardless of their CD4 cell count. After enrollment, some patients started to exhibit viral blips and/or a slight loss of CD4 T cells [57, 58].

The samples collected at PHI M0 (n = 126) and PHI M6 (n = 35) were from subjects enrolled in the French ANRS C06 PRIMO cohort, who are thoroughly described in [17]. We used plasma leftovers for this study. We used data from the ANRS PRIMO cohort to address the relationship between IP-10 and the amount of infected cells, because viral reservoir size has been extensively studied in these patients from PHI onwards [61–64].

Frozen sera collected on EDTA were obtained from the ACS study, while frozen plasma collected on EDTA was obtained from the ANRS cohorts.

EDTA plasma from HIV/HBV/HCV-seronegative individuals (n = 87) were obtained from *Etablissement Français du Sang* (EFS, Paris, France) for research purposes.

### Non-human primates

All animals were housed in the CEA IDMIT facilities (Center for Infectious Disease Models and Innovative Therapies), Fontenay-aux-Roses, France (permit number A 92-032-02) or the Pasteur Institute, Paris, France (permit number A 78-100-3). All experimental procedures were conducted in strict accordance with the European guideline 2010/63/UE for the protection of animals used for experimentation and other scientific purposes (French decree 2013–118). CEA complies with Standards for Human Care and Use of Laboratory Animals of the U.S. Office for Laboratory Animal Welfare under OLAW assurance number A5826-01. The animal experimentation ethics committee approved all experimental protocols (CETEA-DSV, IDF, France; notification numbers 10-051b and 12–006).

Twenty-three (*Chlorocebus sabaeus*) were infected by intravenous inoculation with 250 TCID_50_ of purified SIVagm.sab92018 [13, 14, 15].

Nineteen rhesus macaques (*Macaca mulatta*) and eleven cynomolgus macaques (*Macaca fascicularis*) were infected i.v. with 50 AID_50_ of an uncloned SIVmac251 isolate (provided by A. M. Aubertin, Université Louis Pasteur, Strasbourg, France). Twenty-nine cynomolgus macaques were inoculated intra-rectally with 5 (n = 10) or 50 AID_50_ (n = 13) of the same uncloned SIVmac251 isolate.

In addition, 3 cynomolgus macaques were treated with AZT (4.5 mg/kg) and 3TC (2.5 mg/kg) subcutaneously twice daily and oral indinavir (60 mg/kg) twice daily. The treatment was initiated as early as 4 hours post-challenge and continued until day 14, when the animals were killed. These animals are described in [29]. We used plasma leftovers collected on D9 and D14.

Blood and intestinal samples were obtained from AGM. Blood and lymph node samples were obtained from Cynomolgus macaques. The latter tissues were used for viral load quantification. Blood and intestinal samples were collected from Rhesus macaques, and the tissues were used to measure cellular gene expression.

### IP-10 and sCD163 assays

Whole blood collected on EDTA was used to prepare plasma or serum. IP-10 and sCD163 concentrations were determined in stored plasma or sera samples (−80°C) by specific enzyme-linked immunosorbent assay, human Quantikine CXCL10 and human CD163 Duoset (R&D Systems, Minneapolis, Minnesota) according to the manufacturers' instruction as previously performed.

### Isolation of CD4+ and CD4- cells from NHP gut tissue

At necropsy (day 65 post-infection), a fragment (5/7cm in length) was collected from each of 4 sections of the intestine (jejunum, ileon, colon and rectum) of 5 SIVmac251-infected rhesus macaques and 5 SIVagm.sab92018-infected AGM. The ileum fragment was not collected from 2 rhesus macaques and 2 AGMs. The fragments were enzymatically dissociated in RPMI culture medium (*Life Technologies*) containing collagenase (Collagenase II-S, *Sigma-Aldrich*) and DNAse (*Sigma-Aldrich*) for 1 h with agitation (80 rpm) at 37°C. Total leukocytes were separated from epithelial/endothelial cells through a Percoll gradient. CD4-positive and -negative leukocytes were purified on *Miltenyi* columns and with a CD4 cell purification kit.

### Cell-sorting by flow cytometry

Cryopreserved lymph node cell samples were stained with LIVE/DEAD Fixable Aqua Dead Cell Stain Kit (Thermo Fisher), then labeled with anti-CD45-PerCP, anti-CD4 Pacific Blue and anti-CXCR3-PE-Cy7. Viable CD45+CD4+CXCR3-, CD45+CD4+CXCR3+, CD45+CD4-CXCR3- and CD45+CD4-CXCR3+ cells were sorted using a FACS Aria cell sorter (BD Biosciences) equipped to handle biohazardous material. Human primary Th1 (CD25neg, CXCR3high, CCR4neg, CCR6neg), Th1/Th17 (CD25neg, CXCR3high, CD161+, CCR6+), Th17 CD161+ (CD25neg, CXCR3neg, CD161+, CCR4+ CCR6+) and Th17 (CD25neg, CXCR3neg, CD161neg, CCR4+ CCR6+) were isolated as described [65, 66] from HIV negative blood cytapheresis.

### Evaluation of gene expression in tissues

Total RNA was extracted and reverse-transcribed as previously described [15]. qPCR (Taqman chemistry) and commercial kits were used to quantify the expression levels of genes of interest (CXCL10 Rh02788358_m1, CD14 Rh03648680_s1, CD68 Hs02836816_g1, CD163 Hs00174705_m1). The expression of each gene was normalized to that of 18S rRNA, and relative expression levels were calculated using the ΔΔ*CT* method. The relative gene expression levels were determined by using as the internal reference the raw value for each gene in rectal CD4+ leukocytes from one rhesus macaque, allowing direct comparison between each species (Fig 7A, 7B, 7D–7F and 7J). Alternatively, relative expression levels were determined by normalizing each value against the raw value of each gene in CD4+ leukocytes enriched from the rectum of each animal. This reduces inter-individual differences and highlights differences between the small and large intestine (Fig 7C, 7G–7I, 7K and 7L).

### HIV-1 DNA, SIVmac RNA and SIVmac DNA assays

HIV-1 DNA load in PBMC was measured in the laboratory of Prof. C. Rouzioux [67]. SIV viremia and SIV DNA load in lymphoid tissues were determined as previously described [15, 28]. The cut-offs for cynomolgus macaques were 12 copies/ml of plasma and 12 copies/million cells, respectively.

### Immunohistochemistry

Fresh jejunum fragments were obtained from SIVmac251-infected cynomolgus macaques within 30 min of necropsy (Day 240 p.i.). These fragments were embedded and snap frozen at optimum cold temperature compound (OCT) and 10 μm frozen sections were stained using unconjugated primary antibodies (CD68 clone KP1 from *Santa Cruz*, IP-10 clone ab47045 from *Abcam*) followed by appropriate secondary antibodies conjugated to Alexa 488 (green), Alexa 568 (red) (Molecular Probes, Eugene, OR). Prior to staining, slides were incubated with 100–200 μL of ice cold methanol and 5% acetic acid, allowed to rest at -20°C for 10 min then washed 3 times with PBS. Confocal microscopy acquisition was performed using a Leica TCS SP8 confocal microscope (Leica Microsystems, Exton, PA). Individual optical slices were collected at 512 × 512 pixel resolution. Image J software were used to assign colors to the channels collected.

### Statistical analysis

(See supplementary materials)

## Supporting Information

S1 Text The supplementary information includes the methods used for the statistical analysis of the parameters evaluated in the present study. The tables show the characteristics of the cohorts as well as the correlations between inflammatory markers and HIV-DNA and IP-10. Supplementary Figures section provides supporting graphical evidence of levels of infected cells in lymph node, viral DNA load in the intestine of infected macaques, IP-10 gene expression profile in human Th subsets and IP-10 correlation with multiple factors.
(DOC)
