# Supplementary material for: Elevated Basal Pre-infection CXCL10 in Plasma and in the Small Intestine after Infection Are Associated with More Rapid HIV/SIV Disease Onset
Source: PLoS Pathog. 2016 Aug 10;12(8):e1005774. doi: 10.1371/journal.ppat.1005774 (PMC4980058; doi:10.1371/journal.ppat.1005774)
Supplement: S1 Text — The tables show the characteristics of the cohorts as well as the correlations between inflammatory markers and HIV-DNA and IP-10. Supplementary Figures section provides supporting graphical evidence of levels of infected cells in lymph node, viral DNA load in the intestine of infected macaques, IP-10 gene expression profile in human Th subsets and IP-10 correlation with multiple factors. (DOC) [file ppat.1005774.s001.doc]

**S1 Text**

**Supplementary information**

**Statistical analysis**

Continuous variables from the human cohorts were reported as medians and 25^th^ to 75^th^ percentiles (interquartile range, IQR). They were compared across groups by using the Kruskall-Wallis test for multiple group comparisons, or Wilcoxon non-parametric tests for two-group comparisons. Logistic regression was used identify factors associated with rapid progression (RP, <350 CD4/mm^3^ by M12 post-seroconversion). The factors investigated included the CD4 T cell count and the IP-10 level before HIV-1 infection, as well as the CD4 T cell count, viremia and the IP-10 concentration at all the studied time points after HIV-1 infection and seroconversion (PHI, M3 and M6). Correlations between IP-10 levels and CD4 T cell counts, viremia and plasma soluble markers, as well as between blood IP-10 concentrations and the expression levels of genes of interest in tissues were evaluated by calculating the Pearson correlation coefficient or Spearman’s correlation coefficient, depending on the normality of the data distribution and the group size. A P value <0.05 was considered significant. All analyses were done with Stata 12 software (StataCorp, College Station, Texas) or GraphPad PRISM 6 (GraphPad software, La Jolla CA). Plots and graphs were designed with GraphPad PRISM 6 (GraphPad software, La Jolla CA).

**Table A. Characteristics of the patients from the Amsterdam Cohort Studies on HIV/AIDS included in our study.** IQR=interquartile range, Pre-inf.=pre-infection levels, *closest measurement to date of SC, within the interval -24 months/-3 months before SC, SC= date of seroconversion defined by ACS (see Methods), PHI=primary HIV-1 infection, RP=rapid progressor (<350 CD4/mm^3^ by M12 post SC).

|  | **Total**  **(n=136)** |
| --- | --- |
| Male, n(%) | 118 (86.8) |
| Age (years), median (IQR) | 35 (30-40) |
| CD4 *pre-inf., cells/mm^3^  n (%)  Median (IQR) | 76 (55.9)  895 (715 – 1 205) |
| CD4 at PHI, cells/mm^3^  n (%)  Median (IQR) | 16 (11.8)  620 (435 – 880) |
| CD4 at SC, cells/mm^3^  n (%)  Median (IQR) | 70 (51.5)  835 (620 – 1 160) |
| Viremia at PHI, log copies/ml  n (%)  Median (IQR) | 35 (25.7)  5.50 (4.17 – 6.53) |
| Viremia at SC, log copies/ml  n (%)  Median (IQR) | 106 (77.9)  4.70 (3.76 – 5.26) |
| RP, n (%) | 35 (25.7) |
| Year of SC  1985-1989  1990-1995  1996-2000  2001-2007 | 47 (34.6)  58 (42.6)  16 (11.8)  15 (11.0) |

**Table B. Logistic regression analysis of parameters potentially associated with rapid disease progression.** Univariate analysis of each parameter (CD4, RNA, IP-10 and sCD163) in patients enrolled in the ACS. The values of each parameter were divided into categories. For IP-10 and sCD163, the categories were based on the median. Before infection and at M3 post-SC, patients with plasma IP-10 concentrations above the median had an increased risk (0R>1) of rapid progression. OR=odd ratio, RP=rapid progressor (<350 CD4/mm^3^ by M12 post SC), M3=3 months post SC (SC defined in Methods), pre-inf.=pre-infection.

|  | **N** | **RP, n(%)** | **Crude OR (95% CI)** | **P value** |
| --- | --- | --- | --- | --- |
| IP-10 pre-inf.  pg/ml  ≤128  ≥129 | 43  44 | 7 (16.3)  17 (38.6) | 1  **3.24 (1.18-8.91)** | **0.01** |
| sCD163 at pre-inf  pg/ml  ≤370  ≥370 | 43  44 | 11 (25.6)  12 (27.3) | 1  1.09 (0.42-2.83) | 0.86 |
| IP-10 at M3 post SC  pg/ml  ≤277  ≥278 | 63  63 | 11 (17.5)  22 (34.9) | 1  **2.54 (1.10-5.83)** | **0.02** |
| sCD163 at M3 post SC  pg/ml  ≤537  ≥537 | 62  62 | 17 (27.41)  14 (22.58) | 1  0.77 (0.34-1.74) | 0.54 |
| CD4 at M3 post SC  cells/mm^3^  ≤500  501-800  ≥801 | 41  48  39 | 25 (61)  7 (14.6)  1 (2.6) | **9.15 (3.31-25.33)**  1  0.15 (0.02-1.31) | **P<0.001** |
| Viremia  at M3 post SC,  log RNA copies/ml  ≤4.00  4.01-5.00  5.01-6.00  ≥6.01 | 32  60  35  3 | 8 (25)  14 (23.3)  10 (28.6)  1 (33.3) | 1.10 (0.40-2.97)  1  1.31 (0.31-3.39)  1.64 (0.14-19.50) | 0.94 |

**Table C. Correlations between IP-10 and markers of immune activation in blood before HIV-1 infection.** Markers of immune activation (KI67, CD38, HLADR and CD70 expression) were assessed by flow cytometry from cryopreserved peripheral blood cells of individuals enrolled in the ACS cohorts, who were selected in this study. We analysed if IP-10 and these markers of immune activation were correlated before infection (<24 months).

| N=10 | **Spearman’s rho** | **P value** |
| --- | --- | --- |
| IP-10  % KI67+ CD4+ T cells | 0.04 | 0.90 |
| IP-10  % CD38+ CD4+ T cells | 0.36 | 0.31 |
| IP-10  % HLADR+ CD4+ T cells | 0.03 | 0.91 |
| IP-10  % CD38+ HLADR+ CD4+ T cells | 0.14 | 0.70 |
| IP-10  % CD70+ CD4+ T cells | - 0.21 | 0.57 |
| IP-10  % KI67+ CD8+ T cells | - 0.04 | 0.90 |
| IP-10  % CD38+ CD8+ T cells | 0.12 | 0.74 |
| IP-10  % HLADR+ CD8+ T cells | - 0.18 | 0.61 |
| IP-10  % CD38+ HLADR+ CD8+ T cells | - 0.18 | 0.63 |
| IP-10  % CD70+ CD8+ T cells | - 0.18 | 0.61 |

**Table D. Correlations between HIV DNA loads and inflammatory blood soluble factor concentrations during primary HIV-1 infection.** These soluble factors were previously quantified by luminex in plasma of HIV-infected patients diagnosed during primary HIV-1 infection (Liovat AS et al). We analysed if each of these factors were correlated with the amount of infected cells in blood determined by the total HIV DNA loads (<24 months).

| N=45 | **Spearman’s rho** | **P value** |
| --- | --- | --- |
| IL1-beta | 0.15 | 0.32 |
| IL6 | 0.05 | 0.74 |
| IL12p70 | 0.05 | 0.72 |
| **IL18** | **0.3** | **0.045** |
| TNF-alpha | 0.26 | 0.08 |
| IFN-gamma | 0.13 | 0.39 |
| IL2 | 0.21 | 0.16 |
| IL9 | 0.07 | 0.78 |
| IL15 | -0.01 | 0.92 |
| IL17 | 0.28 | 0.06 |
| sIL2R-alpha | 0.14 | 0.36 |
| sTRAIL | 0.17 | 0.29 |
| Fractalkine | 0.09 | 0.56 |
| IL8 | 0.27 | 0.07 |
| MCP3 | 0.2 | 0.18 |
| MIP1a | 0.08 | 0.58 |
| MIP1b | 0.11 | 0.46 |
| MDC | -0.04 | 0.81 |
| **Rantes** | **-0.4** | **0.007** |
| FGF2 | 0.3 | 0.06 |
| IL7 | 0.02 | 0.89 |
| FLT3L | -0.11 | 0.45 |
| GCSF | -0.05 | 0.74 |
| GMCSF | 0.26 | 0.08 |
| IL10 | 0.2 | 0.19 |
| TGFb1 | 0.15 | 0.33 |
| MCP1 | -0.09 | 0.57 |

**Table E. Characteristics of the chronically HIV-1-infected patients included in this study.**

VIR and cART patients were from the French ANRS C09 COPANA cohort. HIC patients were from the French ANRS C021 CODEX cohort. VIR=viremic and cART-naive, cART=successful cART >24 months, HIC=HIV-1 controllers. IQR=interquartile range, **at the time of IP-10 measurement, *** 2 patients with undetectable values (200 or 400 HIV-1 RNA copies/ml)

| **Patient categories** | **VIR** | **cART** | **HIC** |
| --- | --- | --- | --- |
| N | 121 | 41 | 82 |
| Men, n (%) | 93 (76.2) | 33 (81.5) | 43 (52.4) |
| Age,years,  median (IQR) | 35 (30 – 42) | 37 (32 - 45) | 46 (40 - 52) |
| CD4**, cells/mm^3^  median (IQR)  range | 364 (286 – 543)  127 – 1 044 | 540 (439 - 697)  306 – 1 272 | 651.5 (462 – 875)  251 – 2 142 |
| Viremia **,  log RNA copies/mL  median (IQR)  range  n (%) <50 copies/mL | 4.36 (3.86 – 4.81)  2.10 – 6.15  0 | -  -  39 (100)*** | 1.30 (1.30 – 2.10)  1.00 – 4.43  52 (64.2) |
| IP-10, pg/ml  Median (IQR)  range | 181.6 (87.4-272.4)  7.56 – 1589.9 | 97 (63.4-141.7)  34.1 – 359.9 | 74.7 (52.6 – 156.6)  7.56 – 592.1 |
| Year of collection of samples used for  IP-10 measurement, N, %  2004  2005  2006  2007  2008  2009  2010  2011  2012  2013  2014 | 5 (4.1)  32 (26.3)  51 (41.8)  27 (22.1)  5 (4.1)  1 (0.8) | 1(2.4)  3(7.3)  10(24.9)  17(41.5)  4(9.7) 3(7.3)  3(7.3) | 8 (9.8)  26 (31.7)  21 (25.6)  10 (12.2)  7 (8.5)  10 (12.2) |

**Supplementary Figures**

**Figure A**. **Levels of infected cells and IP-10 gene expression in lymph node cell subsets.**

Peripheral lymph nodes (LN) from 3 viremic cynomolgus macaques (animals presented in Figure 5) were harvested at day 53 post SIVmac infection. Distinct cell subsets were FACS-sorted and total SIVmac DNA (**A**) and IP-10 gene expression levels (**B**) were determined by PCR and Q-PCR, respectively.

**Figure B** **Viral DNA load in the intestine of SIVmac-infected cynomolgus macaques.** Gut fragments from 4 distinct locations (duodenum, ileum, colon and rectum) were harvested at necropsy (Day 21-28 p.i.) from 7 SIV+ animals and total SIVmac DNA were evaluated by QPCR on total leukocytes.

**Figure C** **Correlations between MX1, IFI30 and macrophage-associated gene expressions in the small intestine.** Indicated cellular gene expression levels were evaluated in CD4neg and CD4+ leukocytes from 2 distinct segments of the intestine of SIV+ non-human primates, i.e. rhesus macaques (n=5) and African green monkeys (n=5) on day 65 p.i. Correlation values are represented here.

**Figure D.** **IP-10 gene expression profile in primary human circulating Th subsets from HIVneg donors**. Primary human Th subsets (Th1, Th1 CD161, Th17 and Th17 CD161) were FACS-sorted, following the gating strategy as described [65, 66], from total blood cytapheresis. (**A-D**) Expression of master transcriptions factors associated with Th1 (TBX21, IFNG), Th17 (RORC, IL17A) were determined by real time Q-PCR to confirm the phenotype of these desired sorted cells. (**E**) *IP-10* gene expression. (**F**) Correlation between *IP-10* and *TBX21* gene expressions in IP-10+ Th cells. (**G**) Correlation between *IP-10* and *IFNG* gene expressions in IP-10+ Th cells. (**H**) Correlation between *IP-10* and *RORC* gene expressions in all studied Th cells


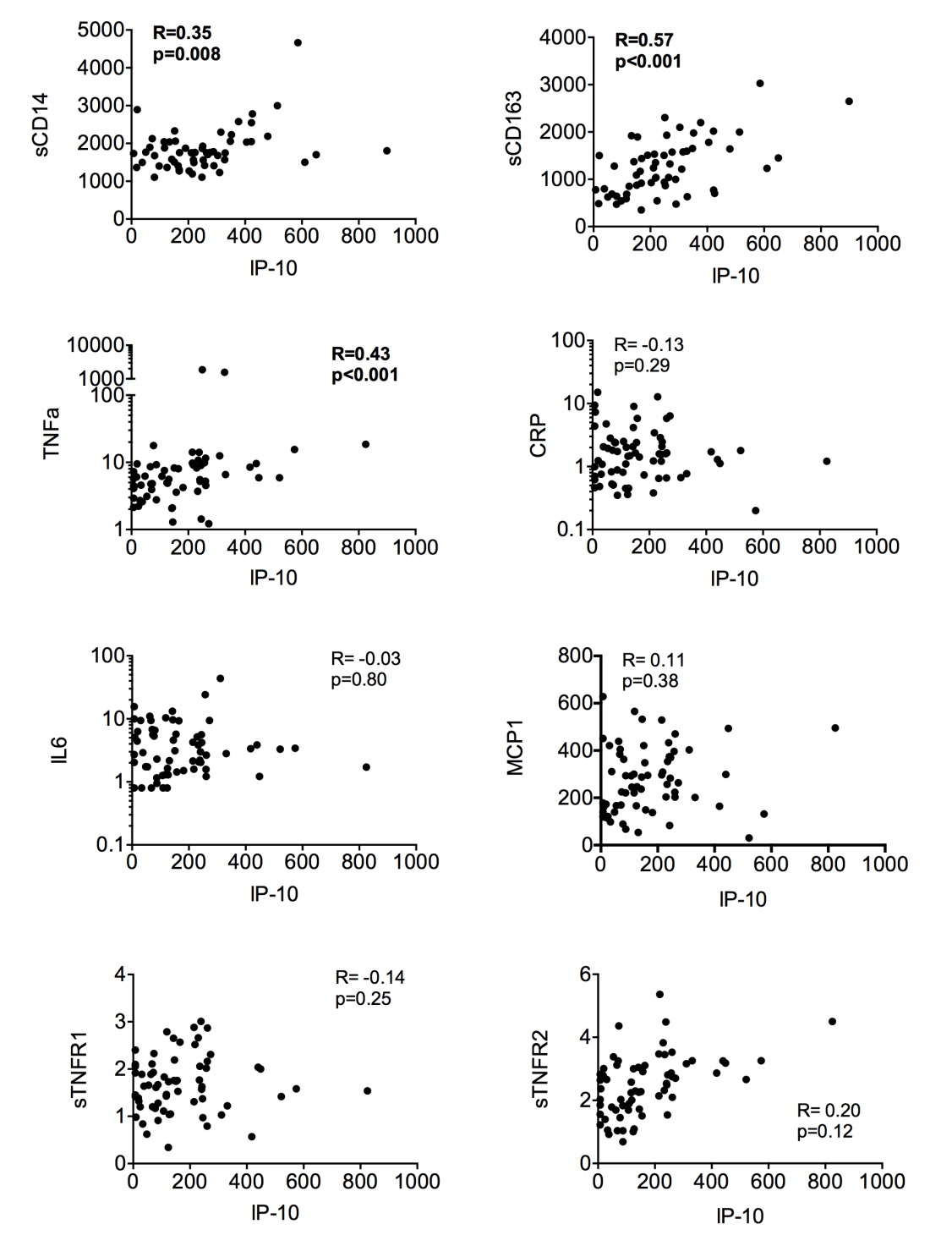


**Figure E.** **Correlations between IP-10 and pro-inflammatory soluble factors in blood during the chronic stage of HIV-1 infection.** Inflammatory soluble factors were previously quantified in chronically HIV-infected patients before cART initatiation (ANRS COPANA cohort) [60]. Here are represented the correlations between IP-10 concentrations in blood with each of these factor
